# Splenic switch-off, a potential novel marker of lack of adenosine response: relationship to heart rate response and demographic factors

**DOI:** 10.1186/1532-429X-17-S1-P92

**Published:** 2015-02-03

**Authors:** Alice Lighton, Marinos Koulouroudias, Filip Zemrak, Charlotte Manisty, James Moon, Ceri Davies, Redha Boubertakh, Mohammed Y Khanji, Ian S Stone, Mark Westwood, Neha Sekhri, Steffen E Petersen

**Affiliations:** Barts and the London School of Medicine and Dentistry, London, UK; Centre for Advanced Cardiovascular Imaging, Willaim Harvey Research Unit, Queen Mary University of London, London, UK; Barts and The London National Institute for Health Research Biomedical Research Unit, London, UK; The Heart Hospital, London, UK

## Background

Haemodynamic response is currently used as a marker of adenosine response during cardiovascular magnetic resonance (CMR) adenosine stress perfusion. However, the sensitivity of these scans is reduced by false negatives, some of which are due to inadequate response to adenosine. Blunted adenosine response can be due to a variety of environmental and pharmacological factors, including recent caffeine intake. Splenic blood flow falls in response to adenosine, splenic switch-off (SSO), and may provide a simple visual marker of adequate stress. The aim of this study was to compare the prevalence SSO to haemodynamic response, and assess its relationship to demographic factors.

## Methods

We examined 503 negative CMR adenosine perfusion scans for SSO by visual assessment. As per local protocol, patients were instructed to avoid caffeine 12 hours before the scan. All patients initially received the standard adenosine protocol (140 mcg/kg/min for at least 3 minutes). If the haemodynamic response was inadequate (HR increase < 10 bpm or SBP decrease < 10 mmHg) then the infusion rate was increased up to a maximum of 210 mcg/kg/min following which an intravenous bolus of 0.05mmol/kg of Gadoteric acid was administered (stress); the second bolus was administered at rest. For the purpose of this study, we considered heart rate increase of 10bpm as a positive haemodynamic response. A multivariate regression model was built using stepwise selection, and covariates were included if they satisfied p<0.05.

## Results

Six scans (1.2%) were excluded due to poor quality and 5 (1%) had no visible spleen. 53 of the remaining 492 scans (11.0%) had no SSO.

Age and gender did not differ significantly between those with or without SSO. People without SSO were more likely to be white (p<0.001).

Haemodynamic response was associated with SSO (OR=2.43, p<0.01). White ethnicity reduced odds of SSO (OR= 0.25). However, a significant proportion of people did not have SSO, but had a positive haemodynamic response (66.0%). Similarly, many people who had SSO did not have a haemodynamic response (18.0%) (table 2).

**Table 1:** Univariate and multivariate regression analyses for predicting splenic switch-off.

	Univariate regression		Multivariate regression	
Predictor	Odds ratio	p-value	Odds ratio	p-value
Male gender	0.62 (0.34 - 1.09)	0.10	0.60 (0.32 - 1.10)	0.10
Haemodynamic response	2.61 (1.39 - 4.76)	<0.01	2.43 (1.27 - 4.57)	<0.01
White ethnicity	0.27 (0.15 - 0.48)	<0.001	0.25 (0.14 - 0.46)	<0.001
Asian ethnicity	1.92 (0.89 - 4.76)	0.12		
Age (≥60 vs <60 years old)	0.78 (0.44 - 1.38)	0.40

**Table 2:** Comparison of SSO and haemodynamic response rates (n=492).

	Haemodynamic response	No haemodynamic response
SSO	360 (82%)	79 (18%)
No SSO	35 (66%)	18 (34%)

## Conclusions

SSO is associated with positive haemodynamic response and is less frequently seen in people of white ethnicity. If SSO is a good indicator of adequate adenosine stress, as many as 23% of patients could have a misclassified response by current haemodynamic methods. The diagnostic accuracy of SSO could be compared against coronary angiography. However, the prognostic value of the presence or absence of SSO can only be inferred after the acquisition of outcome data.

## Funding

AL recieved a Rod Flower scholarship from Barts and the London School of Medicine and Dentistry.

